# Measurement of normal patellar ligament and anterior cruciate ligament by MRI and data analysis

**DOI:** 10.3892/etm.2013.906

**Published:** 2013-01-17

**Authors:** HONGPO WANG, CAIHONG HUA, HONGKAI CUI, YUXIA LI, HAIXIA QIN, DONGMING HAN, JUNYAN YUE, CHANGHUA LIANG, RUIMIN YANG

**Affiliations:** 1 Departments of Radiology, The First Affiliated Hospital of Xinxiang Medical University, Henan, Weihui 453100, P.R. China; 2 Gynecology and Obstetrics, The First Affiliated Hospital of Xinxiang Medical University, Henan, Weihui 453100, P.R. China

**Keywords:** magnetic resonance imaging, anterior cruciate ligament, patellar tendon, clinical anatomy

## Abstract

The aim of this study was to obtain geometric data of *in vivo* patellar ligament (PL) and anterior cruciate ligament (ACL) by MRI and to analyze the correlation of the two with body weight, height and gender. A total of 157 cases with normal sagittal images of bilateral PL and ACL were enrolled. The PL and ACL lengths in the images were measured using the Radworks 5.1 application. The intraclass correlation coefficient for the data measured independently by three doctors was 0.997–1.000. In individuals aged 15–24 years, the values of PL and ACL length and the PL to ACL ratio were 43.95±4.25 mm, 38.45±4.62 mm and 1.15±1.09 in males and 42.03±0.94 mm, 36.00±1.06 mm and 1.18±0.1 in females, respectively. In individuals aged 25–64 years, the values in males were 40.99±4.45 mm, 36.06±3.74 mm and 1.14±0.09 and in females were 39.84±0.64 mm, 36.50±0.81 mm and 1.11±0.02, respectively. In individuals aged ≥65 years, the values in males were 41.43±3.08 mm, 36.62±3.44 mm and 1.15±0.09 and in females were 38.94±0.79 mm, 34.36±0.85 mm and 1.13±0.07, respectively. There was a significant difference between PL and ACL length on the same side (P<0.01). The data obtained was stable and repeatable. The present study established a database of PL and ACL length and the ratio of the two measured by MRI.

## Introduction

The morbidity of anterior cruciate ligament (ACL) injury has been reported to be ∼1 per 3,000 per year in the US population (1). For those engaged in football the incidence of ACL injury is ∼60 per 100,000 per year and for skiing athletes it is 70 per 100,000 per year (2). ACL injury may lead to *articularis genus* instability. Reconstruction should take place within 3 months, but if it is not then osteoarthritis may occur after 4–6 months, resulting in joint replacement and a poor quality of life. Therefore, it is important to diagnose and treat the injury early.

Due to the poor self-healing of the ACL, it requires repair by allograft reconstruction, instead of a simple ACL suture. The autologous transplantation is currently the most widely used in clinical practice and achieves good clinical results. Using an arthroscope, ACL reconstruction using the central third patellar ligament (PL) has become the standard surgical treatment of ACL injury (3). Autologous tissue, including the *semitendinosus* and the *tensor fascia latae* muscles, are used to reconstruct the ACL; however, these tissues are not sufficiently strong and relax over time. Therefore, the prospective efficacy is poor. In these surgical methods, the ligament-tendon insertion is reconstructed into the bone; however, the connection of tendon to bone is not reliable, as it becomes loose or avulses. In ACL recovery using the central third PL, bone-patellar tendon-bone (B-P-B) reconstruction allows bone-bone direct healing. The tensile strength of B-P-B is significantly higher than that of other tissues and the bones on both sides of B-P-B provide fixed points of the reconstructed cruciate ligament. The surgical process achieves bone bio-fixation and is regarded as the best option for orthopedists with the clear advantages of safety, accessibility and high strength (4). In clinical practice, a number of factors may lead to surgery failure, including the shortness, injury or pathological changes of the PL, as well as fractures in the operative field or meniscus and articular cartilage injury. A graft that is too long without sufficient fixation may cause graft-tunnel mismatch (5–8); therefore, the geometric data of the ligaments directly affect the graft and reconstruction (8).

Magnetic resonance imaging (MRI) provides good tissue resolution and high spatial resolution, which allows the clear imaging of bones, including the patella, femur and tibia, as well as the ligamental structures of the patellar tendon and ACL, with a clear boundary from peripheral tissue. Rigorous data may be obtained from MRI images using an MRI workstation with a precision of 0.01 mm. This is helpful for obtaining geometric data and determining the state of the *articularis genus* and ligament of the affected limb prior to surgery.

At present, the samples examined in studies of ACL length measurement have mainly been adult cadaver *articularis genus* specimens and the values reported differ greatly. The Physical Investigation Committee of the Chinese Society for Anatomical Science provided only data for ACL length (9) and previous MRI studies were case reports of abnormal PL (10–13), which are not useful for ACL repair surgery. The present study establishes the geometric data of PL and ACL *in vivo* and provides valuable imaging information for ACL reconstruction.

## Materials and methods

### Inclusion criteria of images

Clear MRI images of the PL and ACL were obtained throughout the whole process with intact continuity. There were low-band signals in sequences and faults of PL. The ACL fiber bundles had distinctly visible fiber directions and equal signals, while the dense fiber bundles presented low signals.

### Enrollment of subjects

A total of 157 cases with PL and ACL images were enrolled from October 2004 to March 2007. All individuals were Han Chinese with bilateral *articularis genus* MRI results. There were 79 male cases, aged 15–71 years, with a mean body weight of 64.24±4.98 kg and height of 169.63±6.06 cm and 78 female cases, aged 15–73 years, with a mean body weight of 56.93±4.88 kg and height of 158.73±4.52 cm. This study was conducted in accordance with the Declaration of Helsinki and with approval from the Ethics Committee of the First Affiliated Hospital of Xinxiang Medical University. Written informed consent was obtained from all participants.

### Examination methods

The GE Signa 1.5 Tesla superconductive magnetic resonance and dedicated *articularis genus* surface coil were used in the present study. In the examination, the subject lay supine with their knees placed in a comfortable position and the center of the coil was set at the inferior margin of the patella. The scan parameters were as follows: echo time (TE), 10 msec; repetition time (TR), 575 msec; echo train length (ETL), 2; bandwidth (BW), 20.83; field of vision (FOV), 16; slice thickness (ST), 4 mm; S-interval, 1 mm; frequency coding (Freq), 384; phase encoding (Phase), 256 and excitation number (Nex), 2.

### PL and ACL measurement

**Figure 1.**
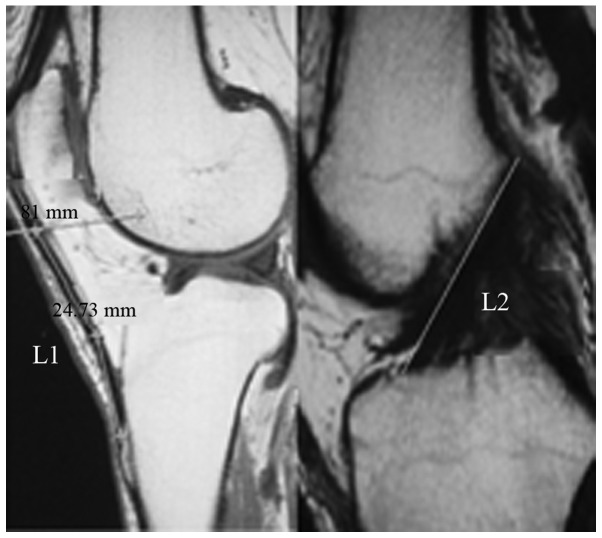
Measurement of the patellar ligament (L1) and the anterior cruciate ligament (L2).

PL length in the oblique sagittal position (L1) was from the lower edge of the patella to the tibial tubercle. In the ACL length (L2) measurement, the starting point was the top of the lateral intercondylar notch in the femoral attachment and the end was the front *facies ossea* of the *eminentia intercondylaris* in the tibial attachment point (L2 line). We calculated the ratio of L1 and L2 (L1/L2; Fig. 1).

Features in the MRI images were measured using the Radworks 5.1 professional workstation. In order to ensure reliability, all data were obtained by doctor A first, then, after one month the length of the left patellar tendon was remeasured independently by doctors B and C.

### Statistical analysis

Data are presented as mean ± standard deviation. Statistical analysis was performed using SPSS 13.0 (SPSS Inc., Chicago, IL, USA). The paired t-test was used in the common index of left and right knee ligaments and the independent samples t-test was applied in the comparison between males and females. Linear regression and linear correlation analyses were used in the dependablity statistics for PL data and body weight, body height and ACL length. The data reliability from the same software was identified by intra-class correlation coefficient (ICC) of the PL length measured by doctors A, B and C. P<0.05 was considered to indicate a statistically significant result.

## Results

### ICC in left patellar tendon length

The left patellar tendon lengths determined by the three doctors were almost entirely credible, with an ICC of 0.80–1.00. The ICC was not <0.997 (Table I).

### L1 and L2 in 157 cases

In the 157 cases of MRI data, the L1 and L2 for the left side of the body were not significantly different from those of the right side (P>0.05; Table II).

### PL and ACL

In the MRI measurements of the left and right knees, the lengths of the PL and ACL in males were significantly greater than those in females (P<0.05; Table III).

### Ratio of L1 and L2 (L1/L2)

There was no significant difference in L1/L2 between the left and the right sides, by paired t-test (P>0.05, Table IV).

### L1, L2 and L1/L2 in different age ranges

The L1 of the young group (aged 15–24 years) was significantly different from those of the post-adolescent (aged 25–64) and senior (aged ≥65 years) groups in male and female cases (P<0.05). However, L2 and L1/L2 did not present a correlation with age (P>0.05; Table V).

### Correlation analysis of L1 with body height, body weight and L2 length

L1 had no significant correlation with body height or weight by linear correlation and regression analysis. However, L1 was significantly correlated with the L2 of the same side (Table VI).

## Discussion

ACL reconstruction using the central third PL under an arthroscope has become the standard surgical treatment for ACL injury. In clinical practice, a number of factors lead to surgery failure, including the shortness, injury or pathological changes of the PL, fractures in the operating field or meniscus and articular cartilage injury. The MRI appearance varies according to the pathological and histological changes of the injury. Ligament data measured by MRI are helpful to detect these abnormalities prior to surgery.

MRI has good tissue resolution and spatial resolution. Chen *et al*(14) confirmed that the 0.2 Tesla field strength of MRI clearly shows the ligament route in a normal knee and a lamellar anatomical cross-section in the oblique coronal plane of a frozen knee ACL. He *et al*(15) verified the clinical value of 0.2 Tesla field strength MRI for PL data. In the present study, all images were achieved using 1.5 Tesla MRI with specific coils for the *articularis genus.* The Radworks 5.1 measuring tool was accurate to 0.01 mm; therefore, our study obtained rigorous data with clinical value.

In the 157 cases, the ICCs were ≥0.997 for the left PL length as measured by three doctors. Landis and Koch (16) suggested that an ICC of 0.00–0.20 is not credible, 0.21–0.40 is generally credible, 0.41–0.60 is averagely credible, 0.61–0.80 has good credibility and 0.80–1.00 is almost entirely credible. Our ACL length result was similar to the value (36.00±0.20 mm) provided by the Physical Investigation Committee of the Chinese Society for Anatomical Science (9), which demonstrates the credibility of the present measurements. We considered that measurement results from Radworks 5.1 did not differ significantly between users and data from a single once-repeated survey using these measuring tools was credible.

In a study of an ACL autograft, Hadjicostas *et al*(17) compared the PL, *semitendinosus* and *gracilis* tendon and identified that the latter two have potential advantages in organism remodeling and regeneration with significant high-density collagen fibers and fibroblasts. The *gracilis* and *semitendinosus* tendons also have good strength; however, they do not form the ligament attachment points with soft tissue-bone fixation. In clinical practice, there are no significant differences between the patellar tendon and the *semitendinosus* tendon (18). Previously, a multicenter study demonstrated that the recovery of knee stability in ACL reconstruction with the patellar tendon is higher (20%) than with the *semitendinosus* and *semimembranosus* tendons (19). Noyes *et al*(20) reported that the tensile strength of the ACL is 100%, the central third B-P-B is 175%, the *semitendinosus* tendon is 75% and the *tensor fascia lata* tendon is 35%, in biomechanics. Toumi *et al*(21) observed that the mechanical stress distribution is asymmetric in the proximal end of the PL and the inside section is stronger than the outside.

At present, adult cadaver *articularis genus* samples have been used in a number of internal studies of ACL length measurement and the values reported are different. The Physical Investigation Committee of the Chinese Society for Anatomical Science provided only the value of the ACL length and previous MRI studies were case reports of abnormal PL. Olszewski *et al*(22) observed that the PL length was 43.33±4.21 mm in certain cases and Huang *et al*(23) observed that the PL length was 31.4–47.3 mm in 18 fresh knee specimens. Yoo *et al*(24) discovered using MRI that the PL length was 40.2±4.2 mm from the lower margin of the patella to the tibial tubercle, the width was 30.3±2.7 mm from the PL to the lower margin of the patella and the thickness was 3.2±0.5 mm. The authors also determined that the width was 24.0±2.8 mm from the PL to the tibial tubercle and the thickness was 5.0±0.7 mm. In the present study, the MRI results of the female PL were similar to the results by Yoo *et al* and the MRI results of the male PL were higher than those of the female; however, they were slightly lower than the measurements made by Olszewski *et al*. A reason for these differences may be that different materials were used. The data in one study was measured by caliper rule with an inherent error. Our study revealed that the PL and ACL lengths of individuals aged 15–24 years were different from those of individuals aged 25–64 years and ≥65 years. This difference may be associated with the physical development of individuals aged 15–24 years, demonstrating the necessity for ligament data from different ages to provide information for ACL reconstruction with allografts. Brown *et al*(25) suggested that PL and ACL length has no significant correlation with body weight, height and gender and that the PL length is significantly correlated with ACL length. The present study discovered that PL and ACL length had no significant correlation with body weight and height and the PL length was significantly correlated with ACL length. However, we observed correlations between gender and PL and ACL length.

One study suggested an ideal ‘equidistance’ status between the tibia and femur attachment points in the graft, which means that the distance between the two points remains fixed during knee flexion and extension (26). However, this theory is controversial and while a number of scholars believe that there is such an isometric section in ACL (27,28), others suggest that the ACL has no absolute isometric fibers (29). If the graft is not isometric, the graft is likely to undergo elongation and relaxation with knee flexion and extension. Penner *et al* suggested that permanent deformation would occur if the elongation of the ligament was >5–6% (30). Therefore, ligament deformation would occur when the length change of the graft is >2 mm in ACL reconstruction. In order to ensure the graft length fit for ACL, obtaining geometric data of the PL and ACL in the same age range and the ratio of the two is necessary for clinicians to determine the surgical program. The present study has provided this information for clinical practice. There was no significant difference of L1/L2 ratio in different ages and genders, respectively.

According to the theory of ‘cruciate ligament as four-bar linkage’ in knee kinematics, the anterior or posterior cruciate ligament is always isometric regardless of the degree of flexion and extension in the physiological range of the *articularis genus*. Maintaining the isometric graft in knee flexion and extension is an important biomechanical principle in the ligament reconstruction and the isometric graft is determined by the tunnel position of the femur and tibia. The location of the bone tunnel is the key factor in ACL reconstruction. The present study identified that the ratio of PL and ACL had no significant differences between the left and right side of the body or between males and females. Under conditions of ACL breakage and knee instability, these data of normal PL and ACL length, and the ratio of PL and ACL in the same age range are helpful to calculate the tunnel length. The tunnel external aperture may be descended to increase the tunnel gradient if the PL is too long. Following the adjustment of the graft tension in the B-P-B, the epactal ligament is likely to be reserved in the tibial tunnel and the bone block will not go beyond the tunnel to affect the screw fixation, since the femoral tunnel length is fixed. As a result, isometric fixation and a stable joint may be achieved.

In summary, MRI was used to obtain geometric parameters of *in vivo* PL and ACL for non-invasive and accurate measurements. The database of PL and ACL lengths and the ratio of the two was established by MRI in Han Chinese subjects of different age ranges. In individuals aged 15–24 years, the values in males were 43.95±4.25 mm, 38.45±4.62 mm and 1.15±1.09 and in females were 42.03±0.94 mm, 36.00±1.06 mm and 1.18±0.11, respectively. In individuals aged 25–64 years, the values in males were 40.99±4.45 mm, 36.06±3.74 mm and 1.14±0.09 and in females were 39.84±0.64 mm, 36.50±0.81 mm and 1.11±0.02, respectively. In individuals aged ≥65 years, the values in males were 41.43±3.08 mm, 36.62±3.44 mm and 1.15±0.09 and in females were 38.94±0.79 mm, 34.36±0.85 mm and 1.13±0.07, respectively. Our study achieves non-invasive measurements in a large sample and supplies image data of the *articularis genus* for use in surgery and sports medicine.

Subjects aged <15 years were not enrolled in the present study due to insufficient numbers; therefore, further study is required to supply the measurement data for that age group.

## Figures and Tables

**Table I.** ICC for the measurements of left patellar tendon length from three doctors.

	Left L1 from doctor B	Left L1 from doctor C
Left L1 from doctor A	1.000	0.997
Left L1 from doctor B		0.997

ICC, intraclass correlation coefficient; L1, patellar ligament length in the oblique sagittal position, from the lower edge of the patellar to the tibial tubercle; L2, anterior cruciate ligament measurement, from the top of the lateral intercondylar notch in the femoral attachment to the front *facies ossea* of the *eminentia intercondylaris* in the tibial attachment point.

**Table II.** Comparison of left and right L1 and L2.

	Left (mm)	Right (mm)
N	157	157
L1	41.15±4.24	41.21±4.23^a^
L2	36.40±4.44	36.38±4.45^a^

a P>0.05, compared with the left side. L1, patellar ligament length in the oblique sagittal position, from the lower edge of the patellar to the tibial tubercle; L2, anterior cruciate ligament measurement, from the top of the lateral intercondylar notch in the femoral attachment to the front *facies ossea* of the *eminentia intercondylaris* in the tibial attachment point.

**Table III.** Comparison of PL and ACL in males and females.

		Males		Females	
	Position	N	Length (mm)	N	Length (mm)
L1	Left	79	42.19±4.22	78	40.10±4.01^a^
	Right	79	42.20±4.23	78	40.20±4.02^a^
	Average	158	42.20±4.21	156	40.15±4.00^a^
L2	Left	79	36.99±4.12	78	35.80±4.68
	Right	79	36.96±4.14	78	35.79±4.69
	Average	158	36.98±4.12	156	35.80±4.67^a^

a P<0.05, compared with males. PL, patellar ligament; ALC, anterior cruciate ligament; L1, patellar ligament length in the oblique sagittal position, from the lower edge of the patellar to the tibial tubercle; L2, anterior cruciate ligament measurement, from the top of the lateral intercondylar notch in the femoral attachment to the front *facies ossea* of the *eminentia intercondylaris* in the tibial attachment point.

**Table IV.** Comparison of L1/L2 ratio between males and females.

L1/L2	Male	Female	Total
Left	1.15±0.09	1.13±0.11	1.14±0.10^a^
Right	1.15±0.09	1.13±0.12	1.14±0.10^a^

a P>0.05, compared with the opposite side. L1, patellar ligament length in the oblique sagittal position, from the lower edge of the patellar to the tibial tubercle; L2, anterior cruciate ligament measurement, from the top of the lateral intercondylar notch in the femoral attachment to the front *facies ossea* of the *eminentia intercondylaris* in the tibial attachment point.

**Table V.** L1, L2 and L1/L2 in different age stages.

	Male				Female			
Age (years)	N	L1	L2	L1/L2	N	L1	L2	L1/L2
15–24 (young)	58	43.95±4.25^a^	38.45±4.62	1.15±1.09	36	42.03±0.94^a^	36.00±1.06	1.18±0.11
25–64 (post-adolescent)	58	40.99±4.45	36.06±3.74	1.14±0.09	78	39.84±0.64	36.50±0.81	1.11±0.02
≥65 (senior)	42	41.43±3.08	36.62±3.44	1.15±0.09	42	38.94±0.79	34.36±0.85	1.13±0.07

a P<0.05, young group compared with the post-adolescent and senior groups. L1, patellar ligament length in the oblique sagittal position, from the lower edge of the patellar to the tibial tubercle; L2, anterior cruciate ligament measurement, from the top of the lateral intercondylar notch in the femoral attachment to the front *facies ossea* of the *eminentia intercondylaris* in the tibial attachment point.

**Table VI.** Correlation analysis of L1 with body height, body weight and L2.

	r-value
Left L1 and height	0.143
Right L1 and height	0.137
Left L1 and body weight	0.038
Right L1 and body weight	0.031
Left L1 and Left L2	0.672^a^
Right L1 and Right L2	0.664^a^

a P<0.01. L1, patellar ligament length in the oblique sagittal position, from the lower edge of the patellar to the tibial tubercle; L2, anterior cruciate ligament measurement, from the top of the lateral intercondylar notch in the femoral attachment to the front *facies ossea* of the *eminentia intercondylaris* in the tibial attachment point.
